# A High Voltage Ratio and Low Ripple Interleaved DC-DC Converter for Fuel Cell Applications

**DOI:** 10.1100/2012/896508

**Published:** 2012-12-23

**Authors:** Long-Yi Chang, Kuei-Hsiang Chao, Tsang-Chih Chang

**Affiliations:** Department of Electrical Engineering, National Chin-Yi University of Technology, No. 57, Section 2, Zhongshan Road, Taiping District, Taichung 41170, Taiwan

## Abstract

This paper proposes a high voltage ratio and low ripple interleaved boost DC-DC converter, which can be used to reduce the output voltage ripple. This converter transfers the low DC voltage of fuel cell to high DC voltage in DC link. The structure of the converter is parallel with two voltage-doubler boost converters by interleaving their output voltages to reduce the voltage ripple ratio. Besides, it can lower the current stress for the switches and inductors in the system. First, the PSIM software was used to establish a proton exchange membrane fuel cell and a converter circuit model. The simulated and measured results of the fuel cell output characteristic curve are made to verify the correctness of the established simulation model. In addition, some experimental results are made to validate the effectiveness in improving output voltage ripple of the proposed high voltage ratio interleaved boost DC-DC converters.

## 1. Introduction

Owing to worldwide energy crisis and awareness of environmental protection in recent years, to seek for substitute energy has become an important issue. Among many substitute energies, solar energy, wind energy, hydroelectric power, biomass energy, and fuel cells are green energies with potential development. As for fuel cells, there tend to have been more and more researches and applications recently. The fuel cell is a clean energy without pollution. Its energy, derived from reversed reaction of electrolyzed water, produces dynamic power. Only water is produced after the reaction; hence, there is hardly any environmental pollution. Fuel cells as a source of power are usually applied to electric hybrid automobiles, distributed electric generation system, and portable and stationary power. Among them proton exchange membrane fuel cells (PEMFCs) are the most commonly used because of the following merits: (1) lower temperature during operation, accordingly leading to rapid turning on and off and rapid reaction to the load change; (2) lower operation pressure, thus with higher safety; (3) easily set in mode system; and (4) lower emission ratio and higher conversion ratio [1–4].

Although the proton exchange membrane fuel cell has the advantages mentioned above, due to its own activation loss, ohmic loss, and concentration loss, the output voltage is lowered as a result of load increase. Namely, the fuel cell lowers the output voltage but raises the output current gradually as the output power rises under the added load. Thus, it is a low-voltage high-current output equipment. If we can transfer the low voltage produced by the fuel cell to high voltage, sending it to DC link, there will be a wider range of application [5–13]. In order to upgrade the fuel cell voltage output to the necessary electricity level and avoid the unsteady voltage caused by load change, it is necessary to adjust the fuel cell energy by means of power electronic technique, thus keeping steady the output voltage.

**Figure 1:**
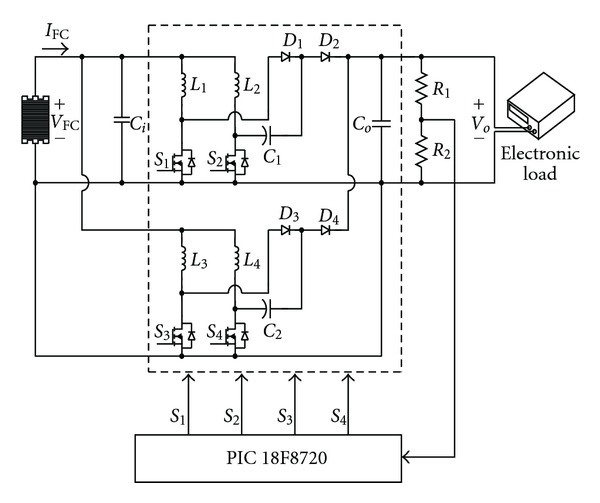
The system of the presented dual interleaved voltage doubler of high voltage ratio converter.

Based on this, presented in this paper is a high voltage ratio interleaved DC-DC converter parallelly connected and further interleaved by means of two sets of voltage-doubler boost converters. So besides the advantages of high voltage ratio converter, also because of the effect of parallel connection, the current is dispersed into four routes, thus lowering the current stress of the switch and inductance. In this way it can withstand the high current output while there is a high load. Through the parallel connection of two sets of converters and controlling their interleaved voltage, it is possible to lower the output voltage ripple ratio.  Figure 1 is the structure of the high voltage interleaved DC-DC converter presented in this paper. The fuel cell provides electricity for the dual interleaved voltage doubler of high voltage ratio converter. Electronic load is used to test the amount of load (light or heavy load); also microcontroller PIC 18F8720 manufactured by Microchip company is used for closed loop control. Because two voltage-doubler boost converters are parallelly connected to interleave the output voltage, the output voltage ripple can be significantly reduced.

## 2. Fuel Cells

There is a great variety of fuel cells; also there are different ways to classify them. The common approach is to classify them according to the various qualities of the electrolyte. Thus, they can be divided into the following six kinds:

1. proton exchange membrane fuel cell, PEMFC,
2. alkaline fuel cell, AFC,
3. phosphoric acid fuel cell, PAFC,
4. molten carbonate fuel cell, MCFC,
5. solid oxide fuel cell, SOFC,
6. direct methanol fuel cell, DMFC.

Among them, the proton exchange membrane fuel cell is the best choice when we choose fuel cells for the source of the applied power because of the following reasons: (1) lower operation temperature, thus it can be rapidly turned on and off; (2) lower operation pressure, hence greater safety; (3) it can be easily set into mode system; (4) lower emission ratio and higher conversion ratio. 

### 2.1. Mold Building of Fuel Cells

As for fuel cells, this paper adopts the NEXA proton exchange membrane fuel cell produced by Ballard Company. The specifications of this proton exchange fuel cell are shown in Table 1 [14].

In building up the proton exchange membrane fuel cell math model, currently there are many simple precise model parameters and calculation formulae being presented and developed [15, 16]. In this paper we refer to the electrochemistry formulae already presented to build up the math model of the proton exchange membrane fuel cell, also within the range of the load current operation simulate the characteristic curve of the output voltage and power rate of the fuel cell [15, 16].

The math model of the proton exchange membrane fuel cell is shown in

(1) $$\begin{array}{c} V_{\text{stack}}=NV_{\text{FC}},\\ V_{\text{FC}}=E_{\text{Nernst}}^{}-V_{\text{act}}-V_{\text{ohmic}}-V_{\text{con}}. \end{array}$$

Therein, *V*_stack_ is the stack output voltage; *N* the number of cells forming the stack; *V*_FC_  the output voltage of the fuel cell; *E*_Nernst_ the output voltage produced by every piece of fuel cell in thermodynamics; *V*_act_ the activation loss; *V*_ohmic_ the ohmic loss; *V*_con_ the concentration loss.

And the thermodynamic output voltage of every piece of fuel cell can be shown as follows.

(2) ENernst=1.229−0.85×10−3(T−298.15)+4.31×10−5T[ln⁡(PH2)+12ln⁡(Po2)].

Therein, *T* is the cell temperature (in Kelvin); *P*_H_2__ is the partial pressures of hydrogen; *P*_O_2__ is the partial pressures of oxygen.

As for activation loss voltage, it can be shown this way:

(3) $$\begin{array}{c} V_{\text{act}}=-\left[\xi_{1}+\xi_{2}\times T+\xi_{3}\times T\times \ln\left(C_{{\text{O}}_{2}}\right)+\xi_{4}\times T\times \ln\left(I_{\text{FC}}\right)\right]. \end{array}$$

Therein, *ξ*_1_, *ξ*_2_, *ξ*_3_, *ξ*_4_ is the parametric coefficient for each cell model; *C*_O_2__ the concentration degree of oxygen in the catalytic interface of the cathode; *I*_FC_ the fuel cell current.

And the respective coefficients of the activation loss are

(4) $$\begin{array}{c} \xi_{2}=0.00286+0.0002\times \ln\left(A\right)+4.3\times 10^{-5}\times \ln\left(C_{{\text{H}}_{2}}\right),\\ C_{{\text{O}}_{2}}=\frac{P_{{\text{O}}_{2}}}{\left[5.08\times 10^{6}\times e^{\left(-498/T\right)}\right]}. \end{array}$$

Therein, *A* is the cell active area, *C*_H_2__ is the liquid phase concentration of hydrogen.

As for ohmic loss voltage, it can be shown as follows:

(5) $$\begin{array}{c} V_{\text{ohmic}}=I_{\text{FC}}\times \left(R_{M}+R_{C}\right). \end{array}$$

Therein, *R*_*M*_ is the resistance coefficient of the membrane, *R*_*C*_ is the resistance coefficient constant to protons transfer through the membrane.

The resistance coefficient of the membrane therein is

(6) $$\begin{array}{c} R_{M}=\rho_{M}\times \frac{L}{A}. \end{array}$$

Therein, *ρ*_*M*_ is the specific resistivity of the membrane to the electron flow, *L* is the thickness of the membrane.

The resistance coefficient of the membrane can be shown to be

(7) ρM={181.6×[1+0.03×(IFCA)        +  0.062×(T303)2×(IFCA)2.5]} /{[λ−0.634−3×(IFCA)×e[4.18×(T−303)/T]  ]}.

Therein, *λ* is the adjustment parameter, the range of which is between 14 and 23.

Concentration loss formula is shown to be

(8) $$\begin{array}{c} V_{\text{con}}=-B\times \ln\left(1-\frac{j}{j_{\max}}\right). \end{array}$$

Therein, *B* is the constant variable depending on the cell type and its working status; *J* is the current density of the cell; *j*_max⁡_ is the maximum current density.

Therein, the current density of the cell is

(9) $$\begin{array}{c} j=\frac{I_{\text{FC}}}{A}. \end{array}$$

**Figure 2:**
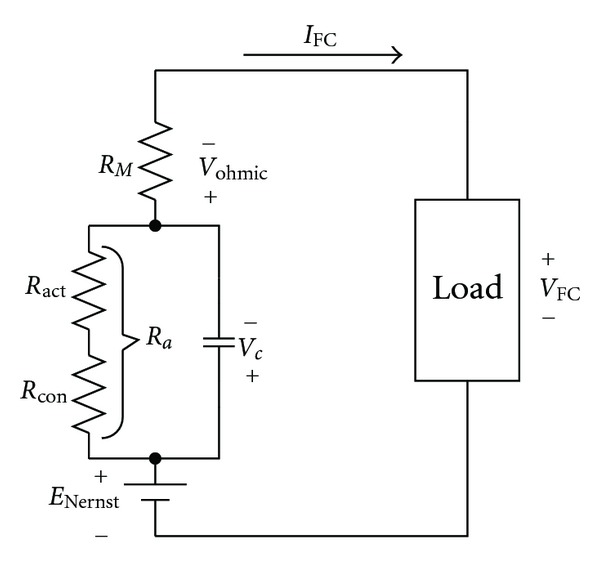
The equivalent circuit of the fuel cell.

Therefore, the equivalent circuit of the fuel cell can be worked up as in Figure 2.

If we take the dynamic response of the fuel cell into consideration, when two different substances come into contact or the load current flows from one end to the other, accumulation of charge is produced on the contact area. In the fuel cell, the layer of change between the electrode and electrolyte (or compact contact face) will accumulate electric charge and energy, whose action is similar to capacitance. So when the load current changes, there will be charge and discharge phenomena happening on the charge layer. Meanwhile, activation loss voltage and concentration loss voltage will be under the influence of transient response, causing delay. But ohmic loss voltage will not be influenced or delayed. We can take this into consideration to let first-order lag exist in activation loss voltage and concentration loss voltage. Thus, its dynamic response equation can be shown to be [15, 16]

(10) $$\begin{array}{c} V_{\text{FC}}=E_{\text{Nernst}}^{}-V_{\text{ohmic}}-V_{c},\\ \frac{dV_{c}}{dt}=\frac{I_{\text{FC}}}{C}-\frac{V_{c}}{\tau },\\ \tau =C\times R_{a}. \end{array}$$

Therein, *τ* is the time constant; *C* is the equivalent capacitance of the system; *V*_*c*_ is the dynamic voltage of the fuel cell; *R*_*a*_ is the equivalent resistance.

The analysis shown above can be used to build up the mathematical model of the proton exchange membrane fuel cell so as to carry on the simulation analysis of the system.

### 2.2. The Simulation of the Fuel Cell

**Figure 3:**
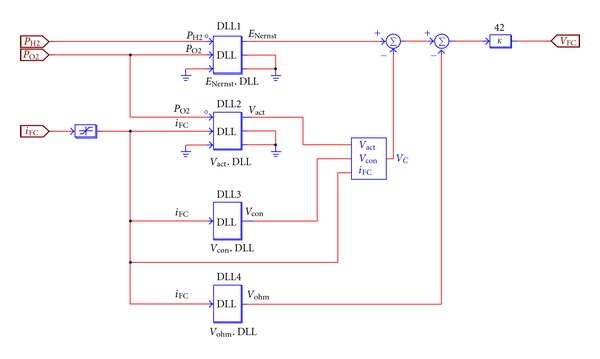
The fuel cell model built up by means of PSIM software.

**Figure 4:**
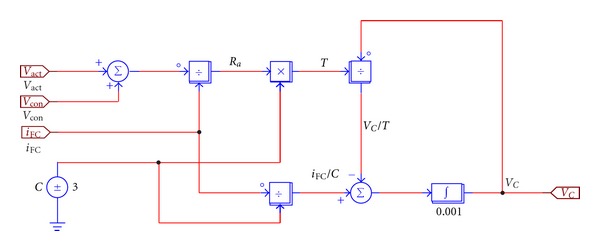
The simulated circuit of capacitance equivalent dynamic action built up by means of PSIM software.

In this paper PSIM simulation software is used to build up the simulated model of the proton exchange membrane fuel cell. Its composition module is shown in Figure 3, in which the upper right increased *k* value is 42, representing the stack amount of the single cell in the cell stack. The simulated circuit of the equivalent capacitance dynamic action is shown in Figure 4.

The DLL in Figure 3 is the dynamic link library of PSIM simulation software. Through software Microsoft Visual C++ 6.0, the necessary DLL file for linking can be set up. By means of Microsoft Visual C++ 6.0, we can make use of programs to write the mathematical formulas in them, saving the trouble of building up numerous inner circuit figures.

**Figure 5:**
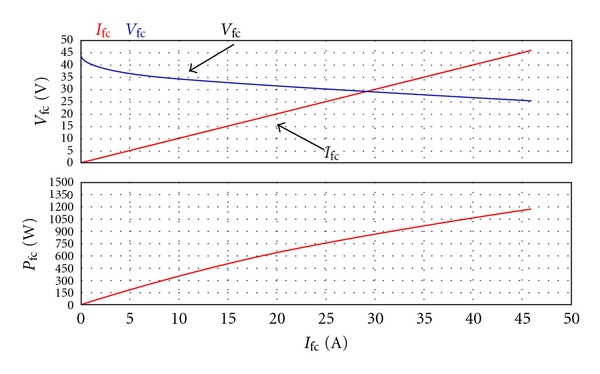
The curve of the fuel cell output by means of PSIM software simulation.

**Figure 6:**
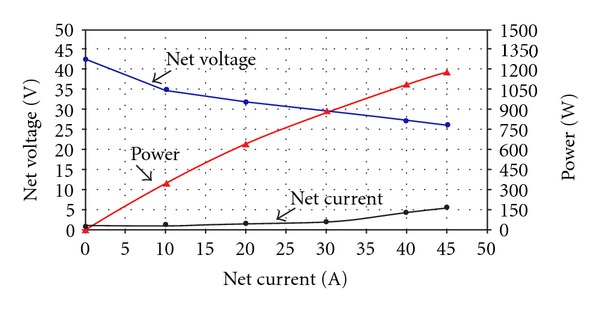
The curve of the actual measuring output of Ballard Co. NEXA fuel cell [14].

After building up fuel cell model, we have its load current operated within fixed rate and value. The hydrogen and oxygen pressures are, respectively, set up at 1 bar. The characteristic curve of the simulated fuel cell output voltage and power rate is shown in Figure 5. The upper part of Figure 5 is the curve of the current and voltage of the fuel cell, while the lower part is the power rate curve. Compared with Figure 6, the actual measuring output curve of Ballard Co. NEXA fuel cell, we can find both of the curves of the output characteristics are closely similar. Only because the curve of Figure 6 is formed by connecting from point to point, it follows that there is slight difference between them.

## 3. Single Set of Voltage-Doubler Boost Converter

**Figure 7:**
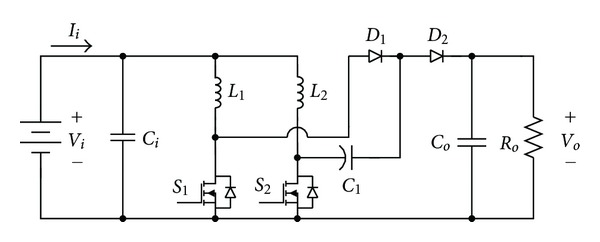
Circuit structure of voltage-doubler boost converter.

**Figure 8:**
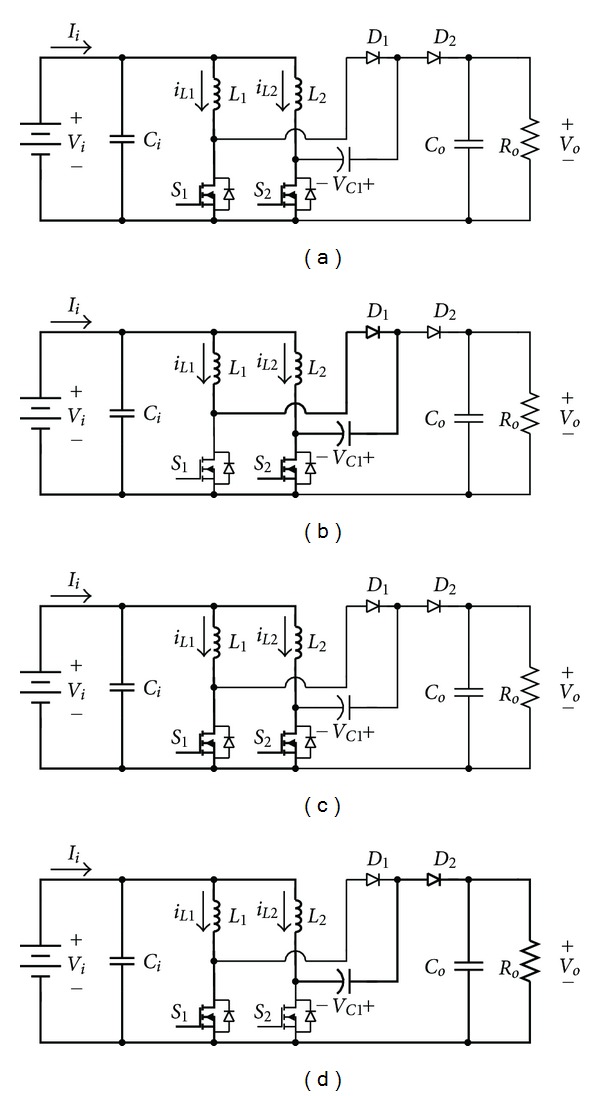
The four switch modes of voltage-doubler boost converter in the duty cycle: (a) model 1, (b) model 2, (c) model 3, and (d) model 4.

Shown in Figure 7 is the circuit structure of single set voltage-doubler boost converter [17, 18]. It is made up of interleaved boost converters with a clamp capacitor *C*_1_. The circuit structure is simple and it can reach the same high voltage ratio with lower duty cycle. Therefore, it can reduce the conduction loss of the switch, to further upgrade the efficiency of the whole converter. The work theorem of the whole circuit can be divided into four operation modes, of which the equivalent circuits are, respectively, shown in Figures 8(a)–8(d).

The equivalent circuits of mode 1 and mode 3 are exhibited in Figures 8(a) and 8(c). In this situation, switches *S*_1_ and *S*_2_ are turned on. Input voltage *V*_*i*_ stays between inductance *L*_1_ and *L*_2_, making the inductance current increase linearly, and begins to deposit energy, and the load current is provided by capacitor *C*_*o*_. The change of the inductance current *i*_*L*1_ and *i*_*L*2_ can be shown in

(11) $$\begin{array}{c} V_{i}=L_{1}\frac{di_{L1}}{dt}=L_{2}\frac{di_{L2}}{dt}. \end{array}$$

Figure 8(b) is the equivalent circuit in mode 2, in which switch *S*_1_ is turned off while *S*_2_ is turned on. The inductance current in forward direction conducts diode *D*_1_. In the meantime inductance *L*_1_ voltage releases energy to clamp capacitor *C*_1_, charging capacitor *C*_1_, while inductance *L*_2_ goes on depositing energy. The change of the inductance current *i*_*L*1_ can be shown in

(12) $$\begin{array}{c} \frac{di_{L1}}{dt}=\frac{V_{i}-V_{C1}}{L_{1}}. \end{array}$$

The equivalent circuit of mode 4 is exhibited in Figure 8(d), in which switch *S*_1_ is turned on and switch *S*_2_ is turned off. The inductance current in forward direction conducts diode *D*_2_. Then inductance *L*_2_ and clamp capacitor *C*_1_ simultaneously release energy to output capacitor *C*_*o*_ and load. The change of inductance current *i*_*L*2_ can be shown in

(13) $$\begin{array}{c} \frac{di_{L2}}{dt}=\frac{V_{i}+V_{C1}-V_{O}}{L_{2}}. \end{array}$$

**Figure 9:**
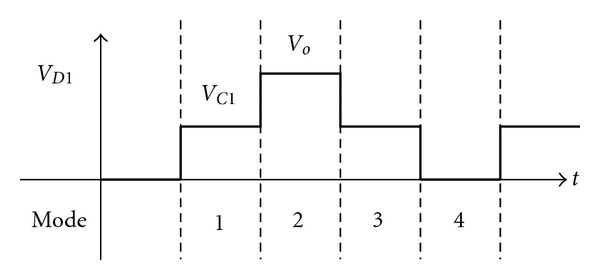
Voltage waveform of diode *D*_1_ under each mode.

Through the analysis of the four modes mentioned above, only *V*_*C*1_ capacitor voltage is an unknown variable. According to circuit structure and KVL theorem, inductance *L*_1_, *L*_2_ and the voltage of diode *D*_1_ plus clamp capacitor voltage *V*_*C*1_ should be zero, and in steady state the average voltage of inductance *L*_1_ and *L*_2_ is zero. Therefore, it is known that the average voltage of *D*_1_ is identical with clamp capacitor voltage *V*_*C*1_. The waveform of *D*_1_ voltage is exhibited in Figure 9, so the clamp capacitor voltage *V*_*C*1_ can be shown in

(14) $$\begin{array}{c} V_{C1}=V_{D1,\;\text{avg}}=\frac{V_{O}}{2}. \end{array}$$

After getting the clamp capacitor voltage, we work out (11)–(13) according to volt-second balance theorem and get (15). Then we carry in (14) to work out (16). Therefore, we can infer that the voltage increase of the converter is shown in (17), in which *T* is the switching cycle, *D* is the duty cycle and *f* is the switching frequency:

(15) $$\begin{array}{c} \frac{V_{i}-V_{C1}}{L_{1}}\times \left(1-D\right)T+\frac{V_{i}}{L_{1}}DT=0, \end{array}$$

(16) $$\begin{array}{c} \frac{V_{i}-\left(V_{O}/2\right)}{L_{1}}\times \left(1-D\right)T+\frac{V_{i}}{L_{1}}DT=0, \end{array}$$

(17) $$\begin{array}{c} V_{O}=\frac{2V_{i}}{1-D}. \end{array}$$

From (17) it is known that voltage-doubler boost converter can reach the same high voltage ratio with a shorter duty cycle. Moreover on account of the added clamp capacitor, the voltage of the switch can be reduced to only half of the output voltage. This can be known from the switch voltage of (18) while operating under mode 2 and mode 4:

(18) $$\begin{array}{c} V_{ds1,\;\max}=V_{C1}=\frac{V_{O}}{2},\\ V_{ds2,\;\max}=V_{C1}=\frac{V_{O}}{2}. \end{array}$$

The output and input power can be shown, respectively, in

(19) $$\begin{array}{c} P_{O}=\frac{{V_{O}}^{2}}{R}, \end{array}$$

(20) $$\begin{array}{c} P_{i}=V_{i}I_{i}=V_{i}\times \left(I_{L1}+I_{L2}\right). \end{array}$$

From (20), assuming *L* = *L*_1_ = *L*_2_, it follows

(21) $$\begin{array}{c} P_{i}=V_{i}I_{i}=V_{i}\times 2I_{L}. \end{array}$$

If there is no power loss of the converter, then *P*_*o*_ = *P*_*i*_ with the following result

(22) Vi×2IL=VO2R=(2Vi/(1−D))2R=4Vi2(1−D)2R,IL=2Vi(1−D)2R,  IL1=IL2=IL.

**Figure 10:**
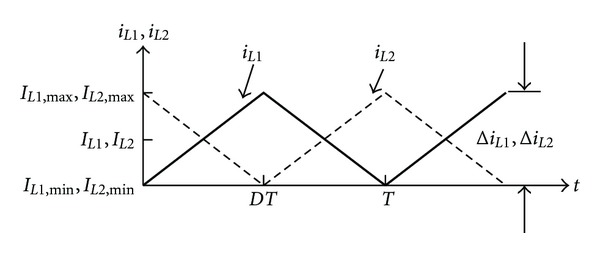
The waveform of the change of inductance current.

The waveform of inductance currents is exhibited in Figure 10, in which though *i*_*L*1_ and *i*_*L*2_ waveforms are in complementary relation, its maximum and minimum inductance current are the same. Hence based on *I*_*L*1_, the related formulae of the maximum and minimum inductance current are, respectively, shown in

(23) IL1, max⁡=IL1+ΔiL12=2Vi(1−D)2R+ViDT2L1,IL1, min⁡=IL1−ΔiL12=2Vi(1−D)2R−ViDT2L1.

The condition on which the converter can be operated in continuous current mode is that *i*_*L*1,min⁡_ and *i*_*L*2,min⁡_ should at least be greater than zero. So the boundary condition of continuous and discontinuous inductance current is

(24) $$\begin{array}{c} I_{L1,\;\min}=0=\frac{2V_{i}}{{\left(1-D\right)}^{2}R}-\frac{V_{i}DT}{2L_{1}}. \end{array}$$

So we get

(25) $$\begin{array}{c} L_{1,\;\min}=\frac{D{\left(1-D\right)}^{2}R}{4f}. \end{array}$$

Because the maximum and the minimum induction currents of inductance *L*_1_ and *L*_2_ are the same, the minimum induction rates derived from *L*_1_ and *L*_2_ are identical. Hence, if the converter is to be operated in the continuous current mode, inductance *L*_1_ and *L*_2_ must at least be greater than or equal to *L*_1, min⁡_.

From the mathematic function *D*(1−*D*)^2^ of (25), it can be observed if *D* value is at 1/3, the mathematic function *D*(1−*D*)^2^ will have the maximum value, which also means the maximum *D* value created by (25) is 1/3. Hence in designing inductance, when *D* as 1/3 is substituted into (25), and let the inductance value derived from calculation be multiplied by surplus value 1.25, it can be assured that the inductance current can really work in the continuous current mode.

The load impedances of so-called light load and heavy load in this paper, are respectively, 2,020 Ω and 450 Ω. So at switching frequency 15 kHz, heavy load duty cycle about 0.85 when it is substituted into (25), the result is that in order to let the current continue under light load, the least inductance should be 6.23 mH, while under heavy load it should be 179 **μ**H. In this paper 260 **μ**H is the option to make it possible to be in continuous current conduction mode under heavy load.

**Figure 11:**
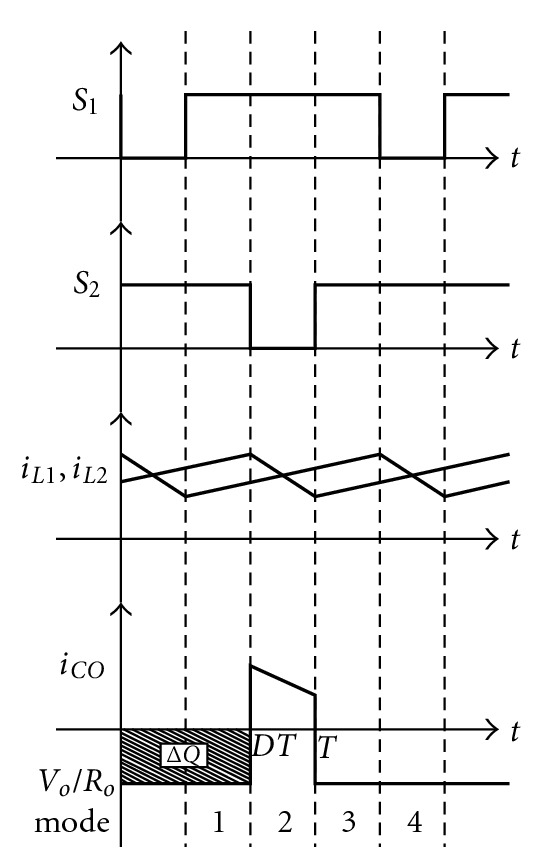
The switch signal, inductance, and capacity waveforms under each operation mode.

The change of output capacitor current is shown in the *i*_*Co*_ of Figure 11. From Figure 11 we know the amount of capacitor electric charge change as

(26) $$\begin{array}{c} \left|\Delta Q\right|=\frac{V_{O}DT}{R_{O}}=C_{O}\Delta V_{O}. \end{array}$$

Then its voltage ripple ratio may be expressed as follows:

(27) $$\begin{array}{c} \frac{\Delta V_{O}}{V_{O}}=\frac{DT}{R_{O}C_{O}}. \end{array}$$

So the result is

(28) $$\begin{array}{c} C_{O}=\frac{D}{R_{O}f\left(\Delta V_{O}/V_{O}\right)}. \end{array}$$

Therefore in the converter, we can decide the size of the capacitor according to the amount of voltage ripple ratio. From (28) it is observed that the output capacity and duty cycle are in linear relation. It means the designed output capacity must be greater than the required capacity with the maximum duty cycle. In this paper voltage ripple ratio is set at 5%. When it is substituted into (28), the output capacity is 2.5 **μ**F. So 150 **μ**F is selected to make the voltage ripple ratio lower than 5%.

By means of the above-described operation mode of the converter, the switch control signal in the circuit, inductance and capacity current waveform can be exhibited in Figure 11, and its input voltage ripple and current ripple can be shown in

(29) ΔIi=(VO/2−ViLn−ViLn)(1−D)T=VO−4Vi2Ln(1−D)T; Ln∈{L1,L2},

(30) $$\begin{array}{c} \Delta V_{Co}=I_{O}DT. \end{array}$$

From (29) it is known that the voltage-doubler boost converter has the advantage of lower input current but the amount of its output voltage ripple is the same as the traditional high voltage converter. Hence in this paper we set forth an ameliorated interleaved voltage-doubler boost converter. By means of the original voltage-doubler boost converter parallelly connected, making output voltage interleaved, so as to reduce output voltage ripple, the flaw of greater output voltage is further ameliorated.

## 4. The Presented Dual Interleaved Voltage Doubler of High Voltage Ratio Converter

**Figure 12:**
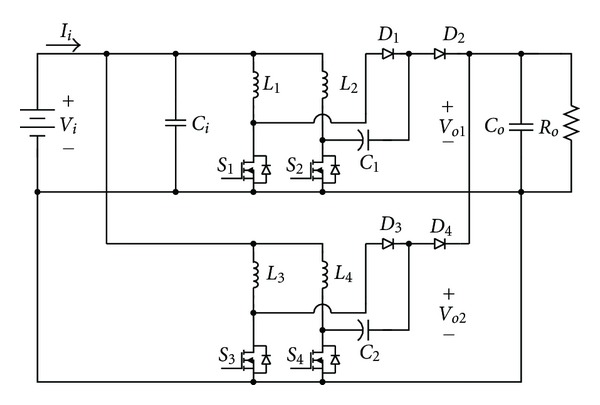
The circuit structure of dual interleaved voltage doubler of high voltage ratio converter.

The circuit structure of the dual interleaved voltage doubler of high voltage ratio converter presented in this paper is shown in Figure 12. By means of parallelly connected original voltage-doubler boost converter to have the two sets of upper and lower voltage mutually interleaved, we can lower its output voltage ripple by controlling one set of their switch control signals to make its output voltage ripple offset that of the other set. In controlling both the upper and the lower sets of switches *S*_1_,  *S*_2_ and *S*_3_,  *S*_4_ to make *S*_1_,  *S*_2_ and *S*_3_,  *S*_4_ switch control phase discrepancy 180° lead to voltage ripple phase displacement, the function of lowering voltage ripple is thus achieved. And because the interleaved switches of these two sets of voltage-doubler boost converters make the input current circuit divide into four routes, thus further lowering the current stress of the inductance and switch, it is possible to withstand the high current of the output of the fuel cell under heavy load. Also it is controlled by microcontroller PIC18F8720. In this way the output voltage can be kept steady at a fixed value. 

**Figure 13:**
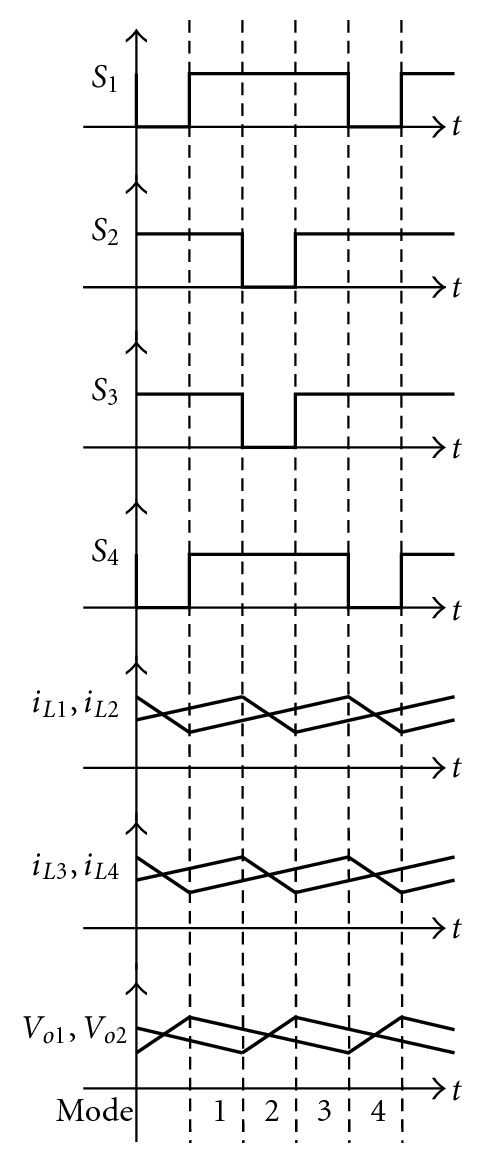
The ripple waveforms of switch control signal, inductance current, and output voltage under each operation mode.

Figure 13 shows the control signal, inductance current, and output voltage ripple waveforms in the circuit. From Figure 13 are observed the output voltage ripples of the two converters *V*_*O*1_ and *V*_*O*2_. Through the phase displacement of the switch control signal, the phase displacement of two sets of voltage ripples is brought about, thus resulting in the effect of lowering the output voltage ripple.

## 5. Experimental Results

In order to prove the feasibility of the dual interleaved voltage doubler of high voltage ratio converter set forth in this paper, a test will be carried on under two different loads. The fuel cell produces output voltage about 26 to 43 V, to be upgraded to 300 V, and the electronic load is, respectively, adjusted at 2,020 Ω (about output power 43 W) and 450 Ω (about output power 200 W) under test.

**Figure 14:**
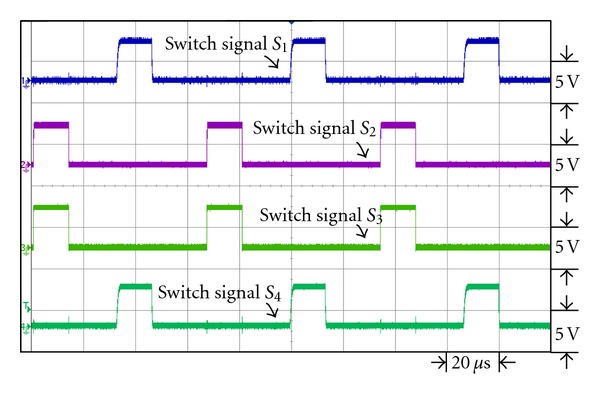
The switch signal waveforms of dual interleaved voltage doubler of high voltage ratio converter.

**Figure 15:**
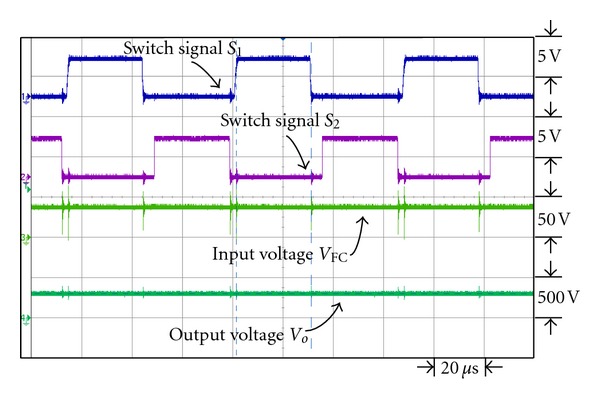
The switch signal and input/output voltage waveforms under output power 43 W.

**Figure 16:**
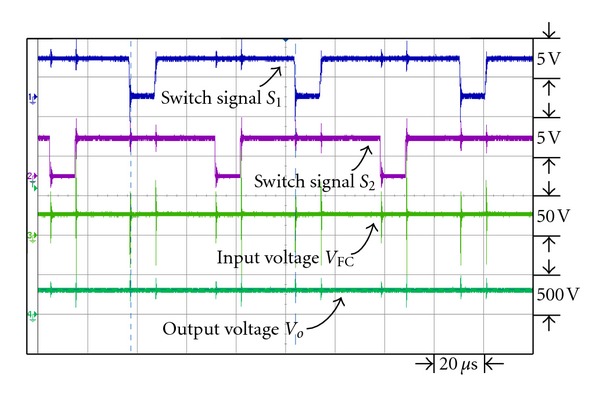
The switch signal and input/output voltage waveforms under output power 200 W.

Figure 14 is the waveforms of the switch signal control in dual interleaved voltage doubler of high voltage ratio converter. Switches *S*_1_,  *S*_3_ and *S*_2_,  *S*_4_ have respective control phase discrepancy 180°. Figures 15 and 16 show the waveforms of switch signal, the waveforms of fuel cell output voltage and output voltage of converter, respectively, under output power 43 W and 200 W. From the figures it is observed that under different loads, by controlling the duty cycle of the switch signal, the output voltage of converter can be kept steady at 300 V.

**Figure 17:**
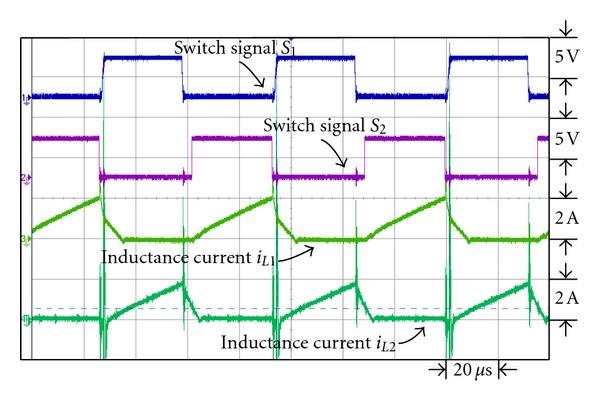
The switch signal, *i*_*L*1_ and *i*_*L*2_ inductance current waveforms under output power 43 W.

**Figure 18:**
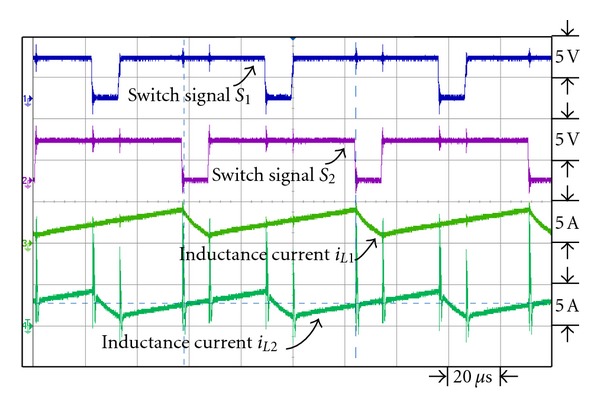
The switch signal, *i*_*L*1_ and *i*_*L*2_ inductance current waveforms under output power 200 W.

Figures 17 and 18 are the waveforms of switch signal and inductance current *i*_*L*1_ and *i*_*L*2_ under respective output power 43 W and 200 W. From the figures it is observed that with the gradual increase of loads, the inductance currents *i*_*L*1_ and *i*_*L*2_ are also on the increase to enable it to work in continuous current mode under higher output power.

**Figure 19:**
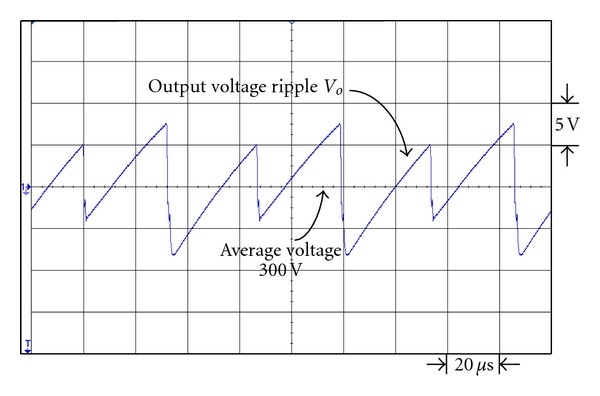
The output voltage ripple waveform of single voltage-doubler boost converter under output power 43 W.

**Figure 20:**
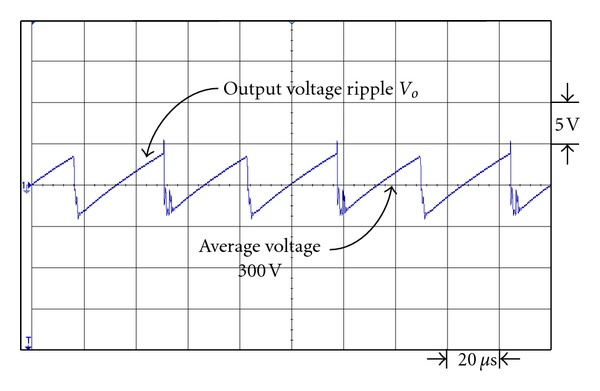
The output voltage ripple waveform of the presented dual interleaved voltage-doubler of high voltage ratio converter under output power 43 W.

Figures 19 and 20 are the respective output voltage ripple waveforms of single set voltage-doubler boost converter and the presented dual interleaved voltage-doubler of high voltage ratio converter. From Figures 19 and 20 it is observed that through comparison we find there is improvement in output voltage ripple waveform. In Figure 19 the peak-to-peak voltage of the single set voltage doubler boost converter is about 15.8 V, while that of the presented dual interleaved voltage doubler of high voltage ratio converter in Figure 20 is about 9.5 V. Their respective voltage ripple ratios are 5.27% and 3.17%.

**Figure 21:**
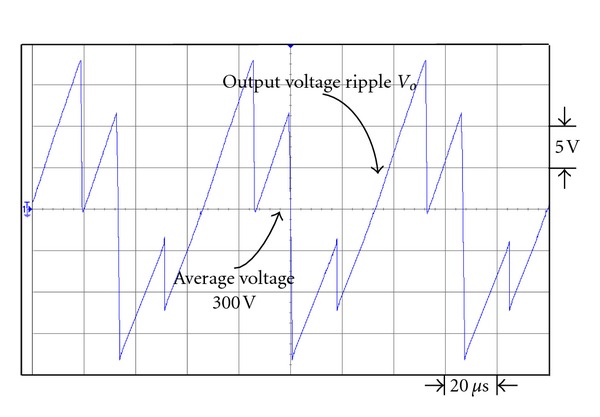
The output voltage ripple waveform of single voltage-doubler boost converter under output power 200 W.

**Figure 22:**
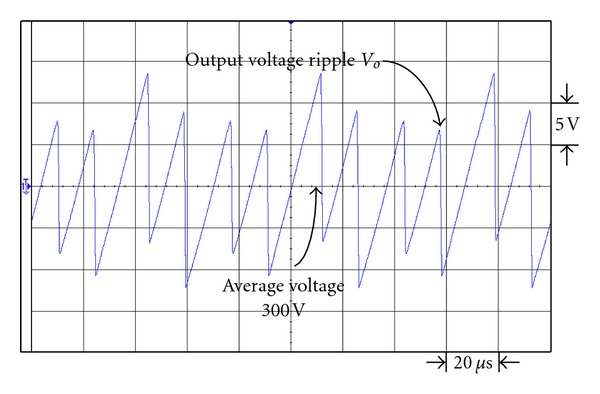
The output voltage ripple waveform of the presented dual interleaved voltage doubler of high voltage ratio converter under output power 200 W.

Figures 21 and 22 are the respective output voltage ripple waveforms of single set voltage doubler boost converter and the presented dual interleaved voltage doubler of high voltage ratio converter. From Figures 21 and 22 it is observed that through comparison we find there is improvement in output voltage ripple waveform. In Figure 21 the peak-to-peak voltage of the single set voltage-doubler boost converter is about 36 V, while that of the presented dual interleaved voltage doubler of high voltage ratio converter in Figure 22 is about 26.25 V. Their respective voltage ripple ratios are 12% and 8.75%. Thus it is proved that the dual interleaved voltage doubler of high voltage ratio converter can improve the flaw of higher voltage ripple ratio of the original single set voltage-doubler boost converter.

## 6. Conclusion

This paper sets forth an ameliorated dual interleaved voltage doubler of high voltage ratio converter to improve the problem of output ripple voltage of single set voltage-doubler boost converter. With two parallelly connected voltage-doubler boost converters to interleave the output voltage ripple, we further lower the output voltage ripple. Not only does it maintain the advantages of voltage-doubler boost converter, but also, owing to the interleaved single set converter with two separate current routes and the two sets of switches of the double voltage booster once again in parallel connection leading to four separate current routes, it is thus possible to further lower the current stress of the switch and inductance. Through test and experiment, this paper proves and confirms the feasibility of the presented dual interleaved converter.

## Figures and Tables

**Table 1:** Specifications of the Ballard NEXA proton exchange membrane fuel cell [14].

Power	Rated power	1200 W
	Operating voltage range	22–50 V_DC_
	Voltage at rated power	26 V
	Current at rated power	46 A
	Startup time	2 minutes

Emissions	Noise	72 dBA
	Water	870 mL/hr

Physical	Dimensions	56 × 25 × 33 cm
	Mass	13 kg

Fuel	Purity	99.99% H_2_ (vol)
	Pressure	0.7–17.2 bar
	Consumption	<18.5 SLPM

## References

[1] Amphlett JC, Mann RF, Peppley BA, Roberge PR, Rodrigues A (1996). A model predicting transient responses of proton exchange membrane fuel cells. *Journal of Power Sources*.

[2] Mann RF, Amphlett JC, Hooper MAI, Jensen HM, Peppley BA, Roberge PR (2000). Development and application of a generalized steady-state electrochemical model for a PEM fuel cell. *Journal of Power Sources*.

[3] Corrêa JM, Farret FA, Canha LN An analysis of the dynamic performance of proton exchange membrane fuel cells using an electrochemical model.

[4] Ali DM A simplified dynamic simulation model (prototype) for a stand-alone Polymer Electrolyte Membrane (PEM) fuel cell stack.

[5] Wai RJ, Lin CY, Chu CC High step-up DC-DC converter for fuel cell generation system.

[6] Wai RJ, Duan RY (2005). High step-up converter with coupled-inductor. *IEEE Transactions on Power Electronics*.

[7] Wai RJ, Liu LW, Duan RY (2006). High-efficiency voltage-clamped DC-DC converter with reduced reverse-recovery current and switch-voltage stress. *IEEE Transactions on Industrial Electronics*.

[8] Thounthong P, Raël S, Davat B (2007). Control strategy of fuel cell and supercapacitors association for a distributed generation system. *IEEE Transactions on Industrial Electronics*.

[9] Thounthong P, Raël S, Davat B (2009). Analysis of supercapacitor as second source based on fuel cell power generation. *IEEE Transactions on Energy Conversion*.

[10] Changchien SK, Liang TJ, Chen JF, Yang LS (2010). Novel high step-up DCDC converter for fuel cell energy conversion system. *IEEE Transactions on Industrial Electronics*.

[11] Thounthong P, Pierfederici S, Martin JP, Hinaje M, Davat B (2010). Modeling and control of fuel cell/supercapacitor hybrid source based on differential flatness control. *IEEE Transactions on Vehicular Technology*.

[12] Shahin A, Hinaje M, Martin JP, Pierfederici S, Rael S, Davat B (2010). High voltage ratio DC-DC converter for fuel-cell applications. *IEEE Transactions on Industrial Electronics*.

[13] Pan CT, Lai CM (2010). A high-efficiency high step-up converter with low switch voltage stress for fuel-cell system applications. *IEEE Transactions on Industrial Electronics*.

[15] Lima LP, Farret FA, Ramos DB PSim mathematical tools to simulate PEM fuel cells including the power converter.

[16] Jia J, Li Q, Wang Y, Cham YT, Han M (2009). Modeling and dynamic characteristic simulation of a proton exchange membrane fuel cell. *IEEE Transactions on Energy Conversion*.

[17] Jang YT, Jovanović MM (2007). Interleaved boost converter with intrinsic voltage-doubler characteristic for universal-line PFC front end. *IEEE Transactions on Power Electronics*.

[18] Pan CT, Lai CM, Cheng MC, Hsu LT A low switch voltage stress interleaved boost converter for power factor correction.

